# Student Health

**Published:** 1859-03

**Authors:** 


### STUDENT HEALTH.

A theological student who was about abandoning his studies in utter discouragement in consequence of declining health, arising from constipation and indigestion, was induced to forego his purpose until he tried what could be done for him by a change of habits, as to eating, sleeping and study. No medicine was advised beyond some half-a-dozen weekly pills. But before he had taken them all he writes " my health is improving, study begins to be a pleasure. I shall ever feel grateful to you for your advice and treatment, to which I attribute my change of health.

There can be no doubt that many young men who might have lived to high distinction, have lost health and life itself from want of timely and judicious advice as to their habits of life, being deterred from seeking that advice from their inability to pay for it. We believe a valuable substitute may be found in our last dollar book on " Health and Disease," in which we mainly strive to show how ordinary ailments may be cured by natural and inexpensive agencies.

